# Comprehensive meta-analysis reveals distinct gene expression signatures of MASLD progression

**DOI:** 10.26508/lsa.202302517

**Published:** 2024-04-02

**Authors:** Ignazio S Piras, Johanna K DiStefano

**Affiliations:** 1 Neurogenomics Division, Translational Genomics Research Institute , Phoenix, AZ, USA; 2 Diabetes and Metabolic Disease Research Unit, Translational Genomics Research Institute , Phoenix, AZ, USA

## Abstract

Metabolic dysfunction-associated steatotic liver disease (MASLD) and its progressive form, metabolic dysfunction-associated steatohepatitis (MASH), pose significant risks of severe fibrosis, cirrhosis, and hepatocellular carcinoma. Despite their widespread prevalence, the molecular mechanisms underlying the development and progression of these common chronic hepatic conditions are not fully understood. Here, we conducted the most extensive meta-analysis of hepatic gene expression datasets from liver biopsy samples to date, integrating 10 RNA-sequencing and microarray datasets (1,058 samples). Using a random-effects meta-analysis model, we compared over 12,000 shared genes across datasets. We identified 685 genes differentially expressed in MASLD versus normal liver, 1,870 in MASH versus normal liver, and 3,284 in MASLD versus MASH. Integrating these results with genome-wide association studies and coexpression networks, we identified two functionally relevant, validated coexpression modules mainly driven by SMOC2, ITGBL1, LOXL1, MGP, SOD3, and TAT, HGD, SLC25A15, respectively, the latter not previously associated with MASLD and MASH. Our findings provide a comprehensive and robust analysis of hepatic gene expression alterations associated with MASLD and MASH and identify novel key drivers of MASLD progression.

## Abstract (precis)

Meta-analysis of hepatic gene expression reveals novel drivers of progression in metabolic dysfunction-associated liver disease (MASLD).

## Introduction

Metabolic dysfunction-associated steatotic liver disease (MASLD), formerly known as nonalcoholic fatty liver disease (NAFLD), is a condition characterized by the accumulation of fat in the liver that can progress to metabolic dysfunction-associated steatohepatitis (MASH), a more severe form of the disease (1, 2). In general, hepatic steatosis attributable to metabolic dysfunction is considered relatively benign, but MASH is linked with greater morbidity and mortality, diminished quality of life, and substantial healthcare costs (3). At present, MASLD is estimated to affect ∼30% of the global population (4, 5, 6), and this figure is projected to rise to 56% by the year 2040 (7). Despite its substantial impact on public health, there are currently no approved pharmacological treatments for MASLD.

Intensive efforts to develop therapeutic agents for MASLD and MASH are presently underway. A comprehensive understanding of the molecular mechanisms driving MASH progression is an important component of this process; however, our current knowledge in this area remains incomplete. One approach to identify potential mechanisms driving the development of MASLD and progression to MASH involves the investigation of hepatic gene expression patterns, and many groups have conducted transcriptomic profiling experiments using liver biopsies obtained from patients spanning the MASLD spectrum (8, 9, 10, 11, 12, 13, 14, 15, 16, 17). Differences in gene expression have identified a core set of genes uniquely associated with advanced fibrosis (8) and discriminated among histological stages on the MASLD spectrum (16, 18, 19, 20). However, findings among these studies have not been consistent, which may reflect differences in methods used to measure gene expression, patient population characteristics, and comparator groups. Furthermore, most reports have been limited by modest sample sizes.

Meta-analysis of gene expression data can provide a more comprehensive and robust view of gene expression patterns than any individual study alone. In addition, this approach can help identify consistent and reproducible gene expression signatures across different studies, potentially leading to new insights into the underlying biology of complex diseases or other biological processes. Thus, to identify characteristics of hepatic gene expression changes associated with MASLD and obtain a deeper understanding of progression to MASH, we performed a meta-analysis of relevant gene expression datasets obtained from the Gene Expression Omnibus (GEO) data repository (21, 22) and sequencing reads archives. We included a total of 1,058 samples from 10 RNA-sequencing (RNA-Seq) and microarray datasets derived from liver tissue, applying strict quality controls and accounting for confounding factors. The integration of the different datasets allowed comparison of more than 12,000 shared genes that were analyzed using a random-effects model implemented in the *GeneMeta* algorithm, a meta-analytical workflow specific for RNA profiling data (23). Furthermore, we conducted a follow-up analysis integrating the meta-analysis results with publicly available datasets from genome-wide association studies (GWAS) and generating coexpression networks from the same datasets included in the meta-analysis. Finally, we projected the relevant coexpression modules in a liver-specific Bayesian regulatory causal network to identify key drivers perturbing the disease-associated networks.

## Results

### Dataset selection

**Figure S1.**
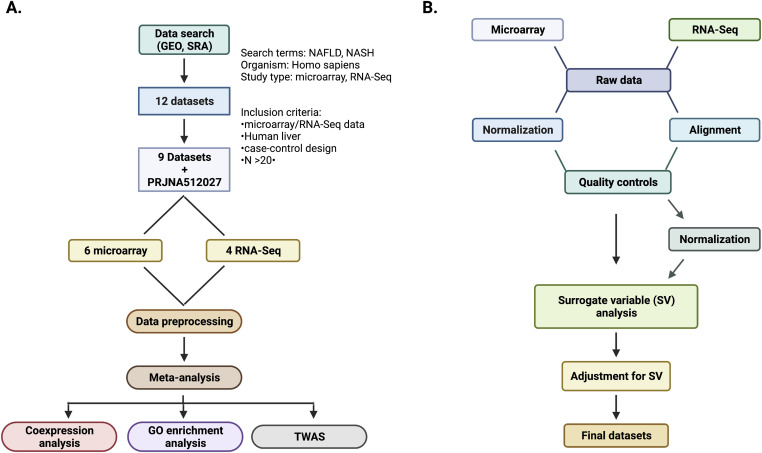
Overview of the study design. **(A)** Workflow of the systematic search of the MASLD and MASH RNA profiling datasets and bioinformatics analysis. **(B)** Details of the preprocessing of the selected RNA profiling datasets.

We performed an extensive search of the Gene Expression Omnibus database (GEO) using “NAFLD,” “NASH,” “steatosis,” “liver fibrosis,” “liver inflammation,” or “fatty liver” as key terms. Twelve datasets were retrieved. We then applied the following inclusion criteria: (1) expression data derived from microarray or RNA-seq; (2) use of RNA that was sourced from human liver tissue; (3) a case-control study design in which MASLD, MASH, and/or a control group (CTL: normal liver) were used; and (4) a total sample size >20. We identified nine datasets meeting these criteria (GEO IDs: GSE135251, GSE126848, GSE130970, GSE167523, GSE83452, GSE61260, GSE48452, GSE33814, and GSE89632), to which we added our previously published RNA-seq dataset (NCBI Bioproject Accession PRJNA512027) (8). Characteristics of these datasets, including sample sizes and post-quality control (QC) checks, are shown in Table 1. These datasets formed the basis for the subsequent meta-analyses and coexpression network analysis (Fig S1A).

**Table 1.** Final sample sizes of all the datasets used in the meta-analysis.

Dataset	Original			After QC			Final sample size	Platform	Reference
	MASH	MASLD	CTL	MASH	MASLD	CTL			
GSE48452	17	9	28	16	8	28	52	Affymetrix microarray	(9)
GSE61260	24	23	62	24	23	61	108	Affymetrix microarray	(13)
GSE83452	104	44	0	104	43	0	147	Affymetrix microarray	(15)
GSE167523	47	51	0	47	50	0	97	Affymetrix microarray	(14)
GSE33814	12	19	13	12	19	13	44	Illumina microarray	(16)
GSE89632	19	20	24	19	20	24	63	Illumina microarray	(10)
GSE135251	155	51	10	155	51	10	216	RNA-Seq	(11)
GSE126848	16	15	26	16	15	26	57	RNA-Seq	(17)
GSE130970	42	36	0	41	34	0	75	RNA-Seq	(12)
PRJNA512027	105	50	36	105	50	36	191	RNA-Seq	(8)

### Differentially expressed genes (DEGs) in MASLD

**Figure 1.**
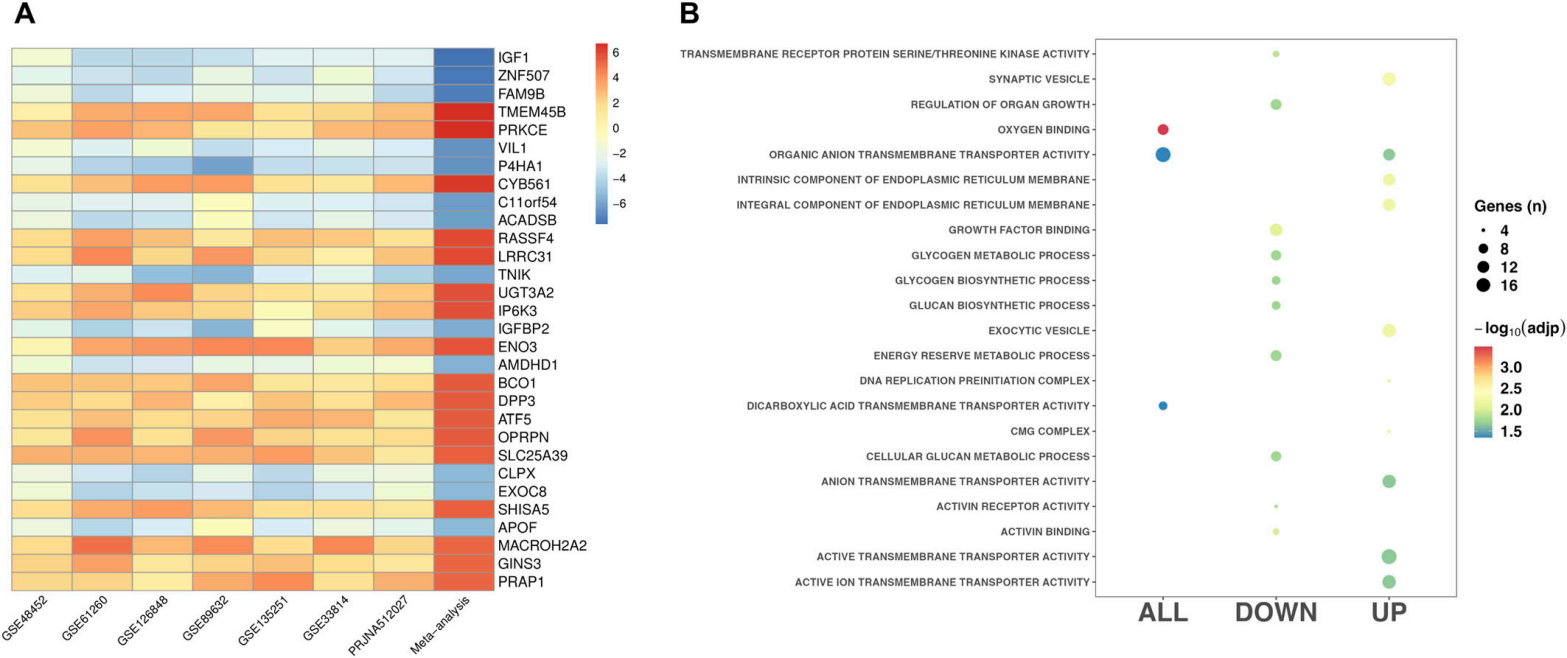
Differentially expressed genes in MASLD. **(A)** The most significant differentially expressed genes identified in the MASLD versus CTL meta-analysis. **(B)** Top GO functional classes (FDR < 0.05) for the differentially expressed genes from the MASLD versus CTL meta-analysis.

The final dataset for the MASLD analysis comprised 516 total samples: 317 MASLD and 199 CTL. Through data harmonization across datasets, we identified 13,376 shared genes. Using a random-effects meta-analysis model, we observed 685 genes showing statistically significant differences in hepatic expression between individuals with MASLD and those with normal liver (Table S1). Of these DEGs, 360 were down-regulated and 325 were up-regulated in those with MASLD. The 30 genes showing the strongest evidence for differential expression are shown in Fig 1A. The five most significant genes were prolyl 4-hydroxylase subunit alpha 1 (*P4HA1*), Ras Association Domain Family Member 4 (*RASSF4*), acyl-CoA Dehydrogenase Short/Branched Chain (*ACADSB*), chromosome 11 open reading frame 54 (*C11orf54*), and transmembrane protein 45B (*TMEM45B*). To gain insights into the biological processes, molecular functions, and cellular components associated with this set of DEGs, we performed pathway analysis using the GO database. Our analysis identified 77 enriched functional classes (Table S2), and the top 10 biological processes for all DEGs, including those that were up- or down-regulated relative to the control group, are shown in Fig 1B. Genes showing reduced expression in MASLD were predominantly associated with activin binding, activin receptor activity, and glycogen biosynthetic processes, whereas those with up-regulated expression in MASLD were associated with processes of exocytosis and ion transmembrane transporter activity.

Table S1 Differentially expressed genes identified in the MASLD versus CTL meta-analysis.

Table S2 Results of the pathway analysis conducted using the differentially expressed genes from the meta-analysis MASLD versus CTL.

### DEGs in MASH

**Figure 2.**
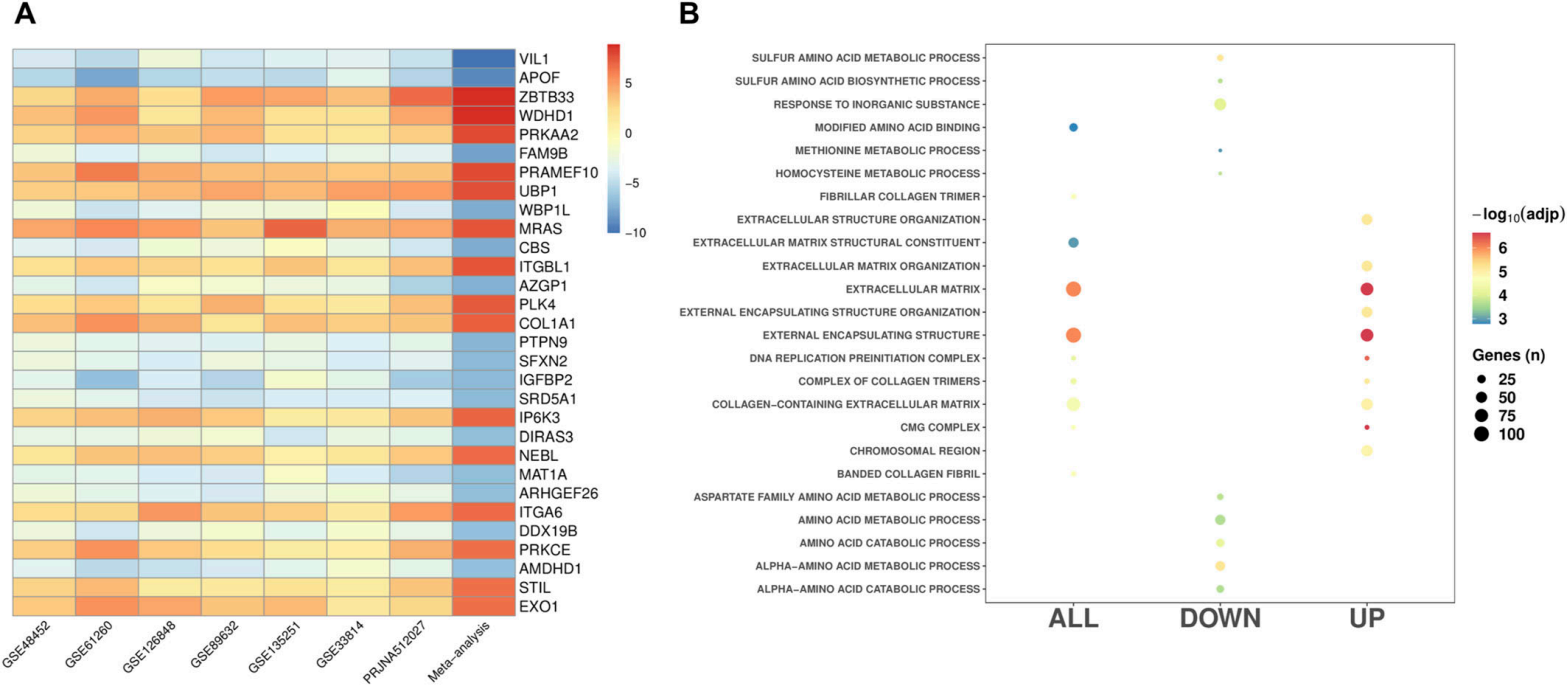
Differentially expressed genes in MASH. **(A)** The most significant differentially expressed genes identified in the MASH versus CTL meta-analysis. **(B)** Top GO functional classes (FDR < 0.05) for the differentially expressed genes from the MASH versus CTL meta-analysis.

To determine gene expression patterns that may be specific to MASH, we compared gene expression between individuals with MASH (*n* = 541) and those with normal liver (*n* = 199). In a comparison of 13,476 shared genes between these two groups, we identified 1,870 genes exhibiting statistically significant differential expression. Among these genes, 1,005 were found to be up-regulated in MASH, whereas 865 were down-regulated (Table S3; Fig 2A). The top DEGs were *P4HA1*, PRAME family member 10 (*PRAMEF10*), protein kinase AMP-activated catalytic subunit alpha 2 (*PRKAA2*), denticleless E3 ubiquitin protein ligase homolog (*DTL*), and exonuclease 1 (*EXO1*), as well as genes such as methionine adenosyltransferase 1A (*MAT1A*) (24), integrin subunit beta like 1 (*ITGBL1*) (25), and insulin-like growth factor binding protein 2 (*IGFBP2*) previously linked with MASLD (26). Pathway analysis revealed 213 significantly enriched GO classes (Table S4). The most significant classes were associated with sulfur and alpha-amino metabolic processes for the up-regulated genes in MASH, whereas the down-regulated genes were primarily associated with extracellular matrix organization (Fig 2B).

Table S3 Differentially expressed genes identified in the MASH versus CTL meta-analysis.

Table S4 Significant GO functional classes for the differentially expressed genes identified in the MASH versus CTL meta-analysis.

### Gene expression patterns associated with MASLD progression

**Figure 3.**
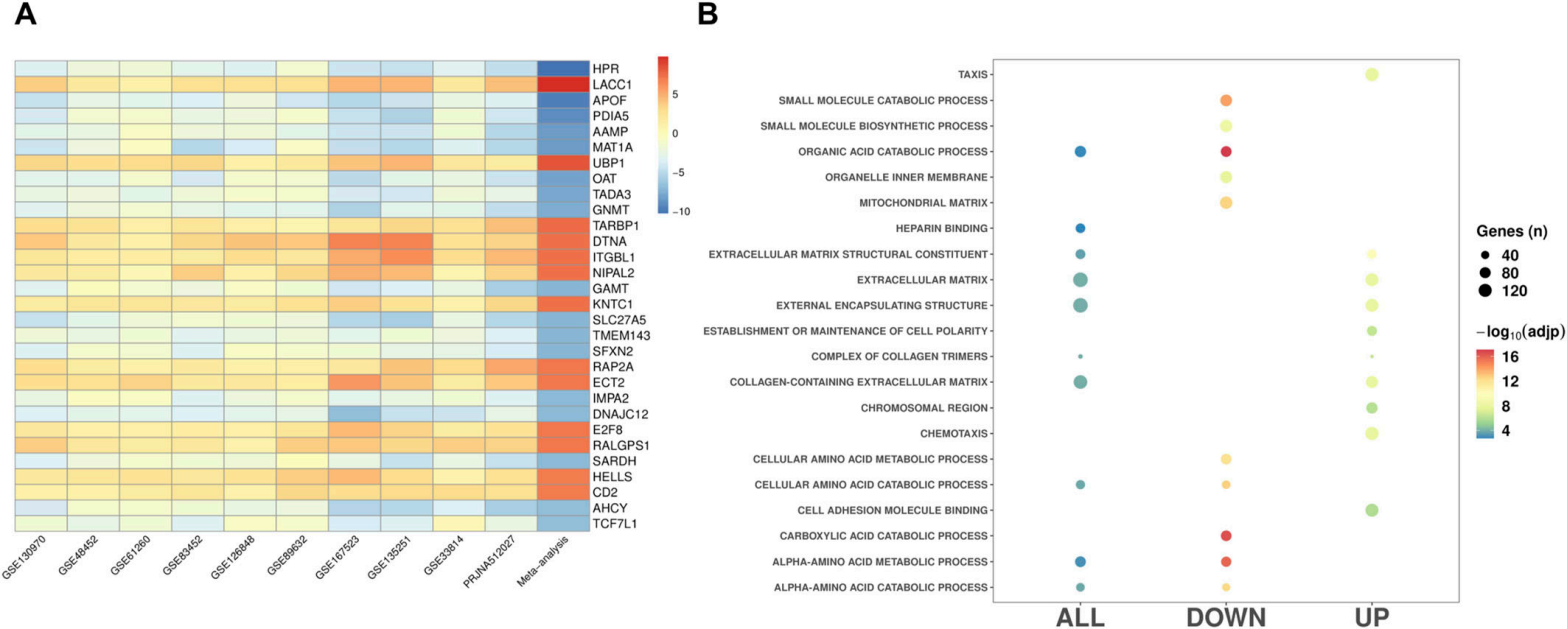
Gene expression patterns associated with MASLD progression. **(A)** The most significant differentially expressed genes identified in the MASH versus MASLD meta-analysis. **(B)** Top GO functional classes (FDR < 0.05) for the differentially expressed genes from the MASH versus MASLD meta-analysis.

We next sought to identify genes involved in MASLD progression by comparing hepatic gene expression values between MASLD and MASH samples. As shown in Table 1, 317 MASLD and 541 MASH samples comprised the final dataset. We observed 12,817 genes shared among these datasets, similar to the number of shared genes identified in the MASLD analysis. However, the random-effects meta-analysis revealed 3,284 genes that were differentially expressed between MASH and MASLD (1,850 up- and 1,434 down-regulated in MASH) (Table S5), which is substantially greater than the MASLD analysis. The genes showing the most significant evidence for differential expression are shown in Fig 3A. Pathway analysis of the 3,284 DEGs revealed 684 significantly enriched functional classes (Table S6). Genes showing up-regulation in MASH relative to MASLD were associated with the extracellular matrix and chemotaxis, whereas those down-regulated in MASH were mainly linked to the catabolic processes of organic compounds, carboxylic acids, and alpha-amino acids (Fig 3B).

Table S5 Differentially expressed genes obtained from the MASH versus MASLD meta-analysis.

Table S6 Significant GO functional classes for the differentially expressed genes detected in the MASH versus MASLD meta-analysis.

### Overlap and shared genes among meta-analyses

**Figure 4.**
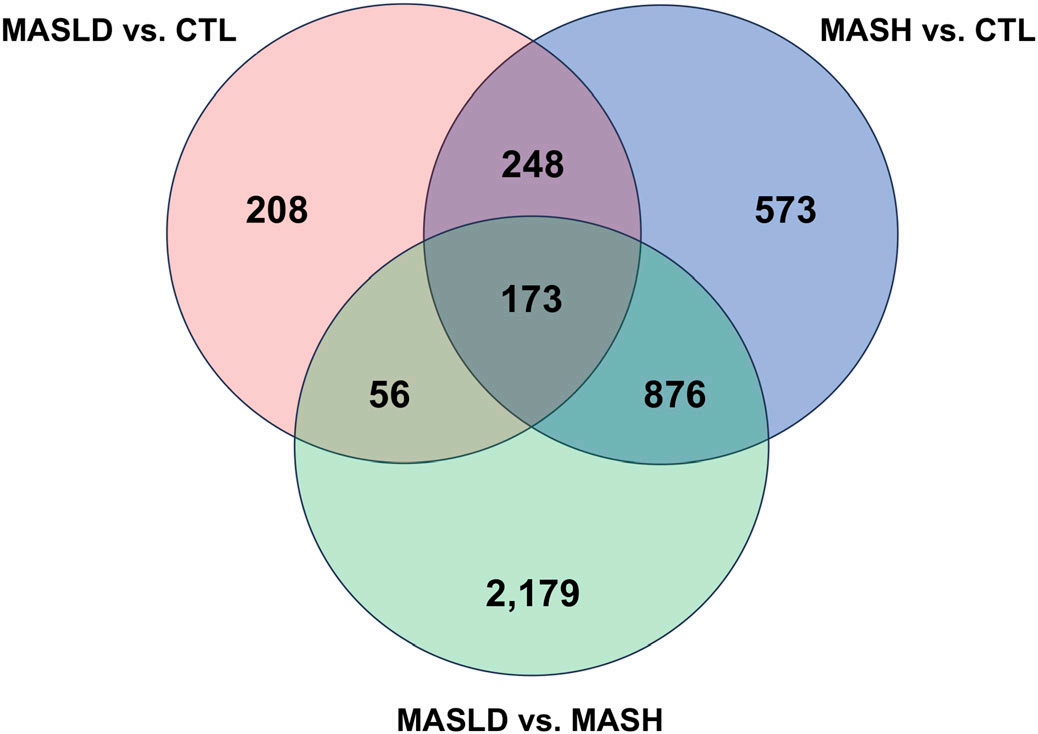
Overlap of differentially expressed genes detected across the three meta-analyses.

When comparing the results from the three meta-analyses, we observed an overlap of 173 genes. In pairwise comparisons of the meta-analysis data, we identified 421 shared genes between the MASLD versus CTL and MASH versus CTL analyses, 1,049 shared genes between the MASH versus MASLD and the MASH versus CTL meta-analyses, and 229 shared genes between MASH versus MASLD and the MASLD versus CTL meta-analyses (Fig 4). The lists of these overlapping genes are presented in Table S7.

Table S7 Overlap of the significant differentially expressed genes among the different meta-analyses.

### Enrichment of GWAS and transcriptome-wide association study (TWAS) genes in the MASLD and MASH meta-analyses

**Figure S2.**
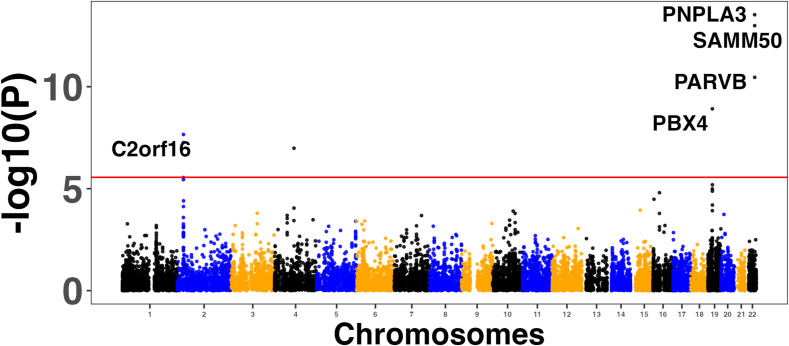
Manhattan plots showing the top five associated genes in the reference (27) genome-wide association studies MAGMA analysis.

**Figure S3.**
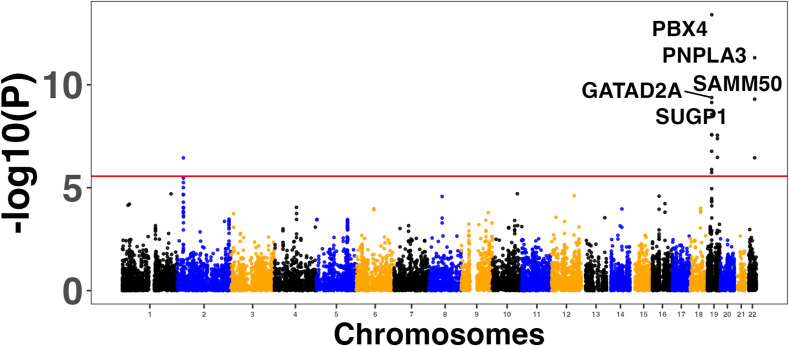
Manhattan plots showing the top five associated genes in the MAGMA analysis conducted on the genome-wide association studies results from reference (28).

**Figure S4.**
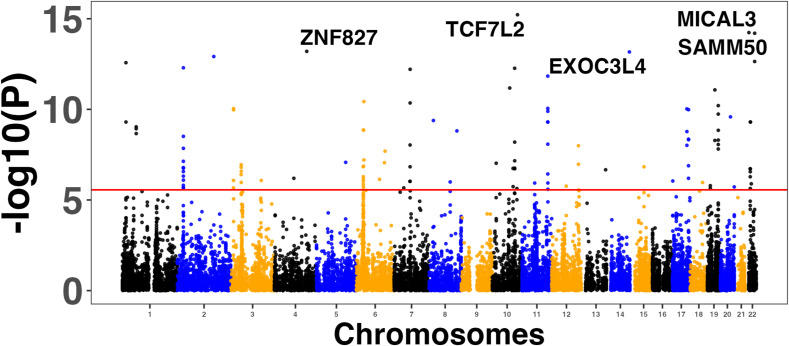
Manhattan plots showing the top five associated genes in the MAGMA analysis conducted on the genome-wide association studies results from reference (29).

**Figure S5.**
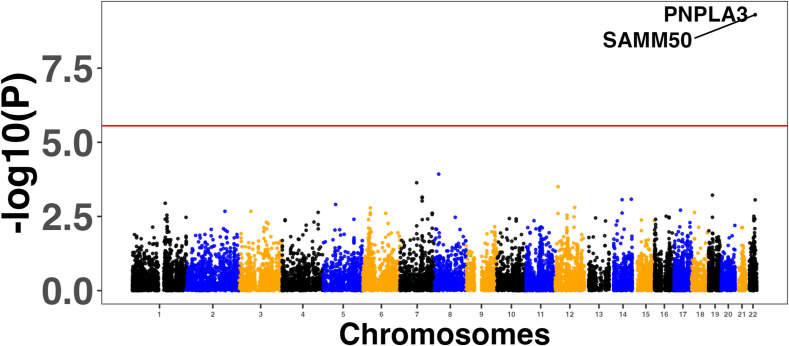
Manhattan plots showing the two associated genes in the MAGMA analysis conducted on the genome-wide association studies results from reference (30).

**Figure S6.**
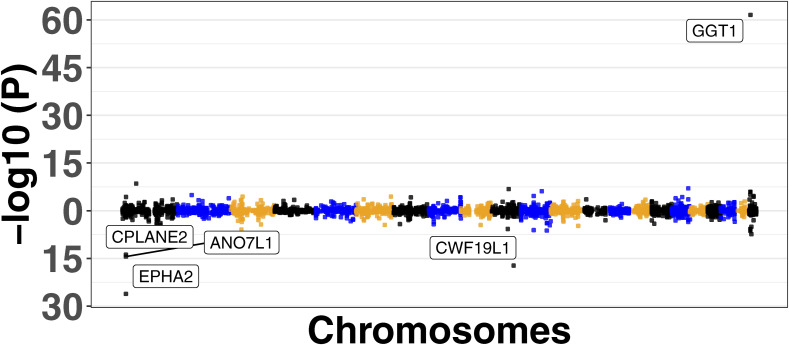
Miami Plot showing the top five genes associated with MASLD in the transcriptome-wide association study analysis from reference (29).

**Figure S7.**
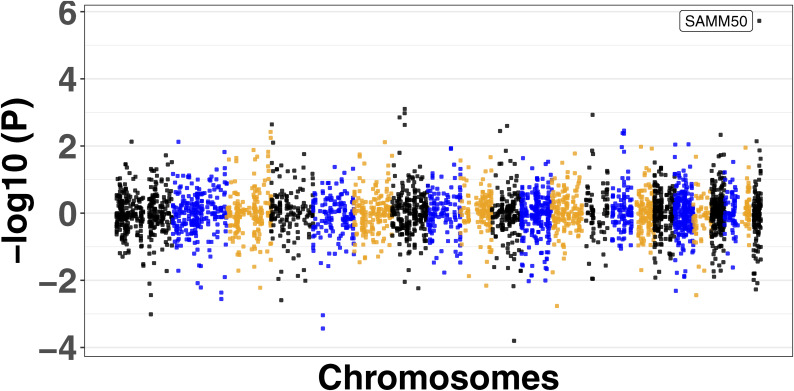
Miami plot showing the associated gene in the transcriptome-wide association study from reference (27).

**Figure S8.**
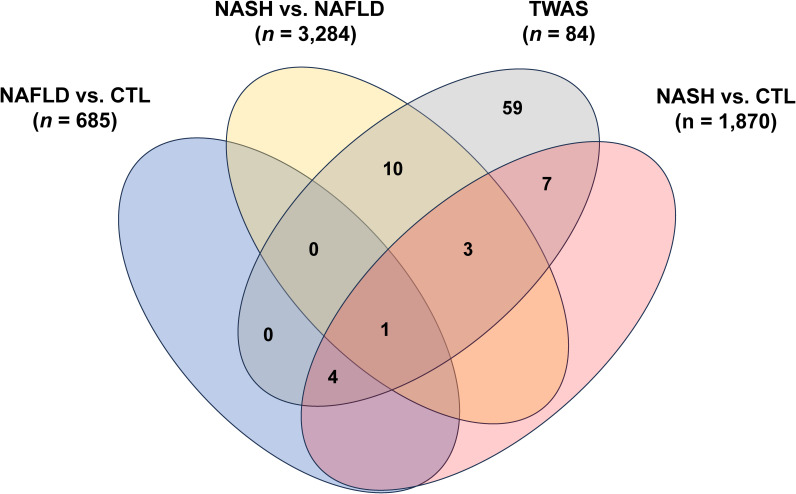
Overlap between the genes identified in the transcriptome-wide association study from reference (29) and the three differential expression comparisons.

We used four large MASLD GWAS (see the Materials and Methods section) for the MAGMA analysis and detected 141 significant signals encompassing a total of 127 unique genes (Table S8). Manhattan plots are shown in Figs S2, S3, S4, and S5. We used this list of genes to run hypergeometric enrichment. Although no significant enrichment was detected (Table S9), we did observe overlap of 47 signals with 36 unique genes identified in the meta-analysis (Table S10). To identify genetic variants associated with gene expression, we conducted a TWAS analysis referencing liver tissue based on four large GWAS studies comprising MASLD patients and unaffected controls (27, 28, 29, 30). These GWAS had a total of 5,187, 439, 82, and 306 SNPs that were significant at the genome-wide level (*P* < 5.0 × 10^−08^). Significant findings from the TWAS were obtained only from the study by reference (29), with 83 genes showing significant associations (Table S11, Fig S6) and one from reference (27) (Table S12, Fig S7). To further investigate the relevance of these 84 significant TWAS genes (adj-p < 0.05), we conducted a hypergeometric enrichment analysis across the meta-analysis. We observed an enrichment of TWAS genes in the comparison between NASH and CTL samples, approaching significance (*P* = 0.034; *P*-adj = 0.103) (Table S13). Overall, five genes overlapped with the MASLD versus CTL meta-analysis, 15 genes with the MASH versus CTL meta-analysis, and 18 genes with the MASLD versus MASH meta-analysis. All the genes were from the TWAS conducted on the reference (29) GWAS data. The Venn diagram in Fig S8 depicts this overlap, and the complete list of overlapping genes can be found in Table S14. Finally, eight of the TWAS genes overlapping with the meta-analysis were also significant in the MAGMA analysis (*L3MBTL3*, *GGT1*, *GSTT2B*, *SLC25A19*, *EPHA2*, *DLG5*, *MRPS7*, and *KLHL18*) (Table S14).

Table S8 Significant genes obtained in the MAGMA analysis from all the MASLD genome-wide association studies.

Table S9 Enrichment results for genome-wide association studies of genes in the three meta-analysis.

Table S10 Overlapping genes between the meta-analsis and the MAGMA results.

Table S11 Significant genes detected from the transcriptome-wide association study using the reference (29) dataset.

Table S12 Significant genes detected from the transcriptome-wide association study using the reference (27) dataset.

Table S13 Enrichment of transcriptome-wide association study genes in the list of differentially expressed genes detected by meta-analysis.

Table S14 Overlap between meta-analysis results and the transcriptome-wide association study computed from the MASLD genome-wide association studies

### Coexpression analysis reveals differentially expressed network modules in MASLD and MASH

**Figure 5.**
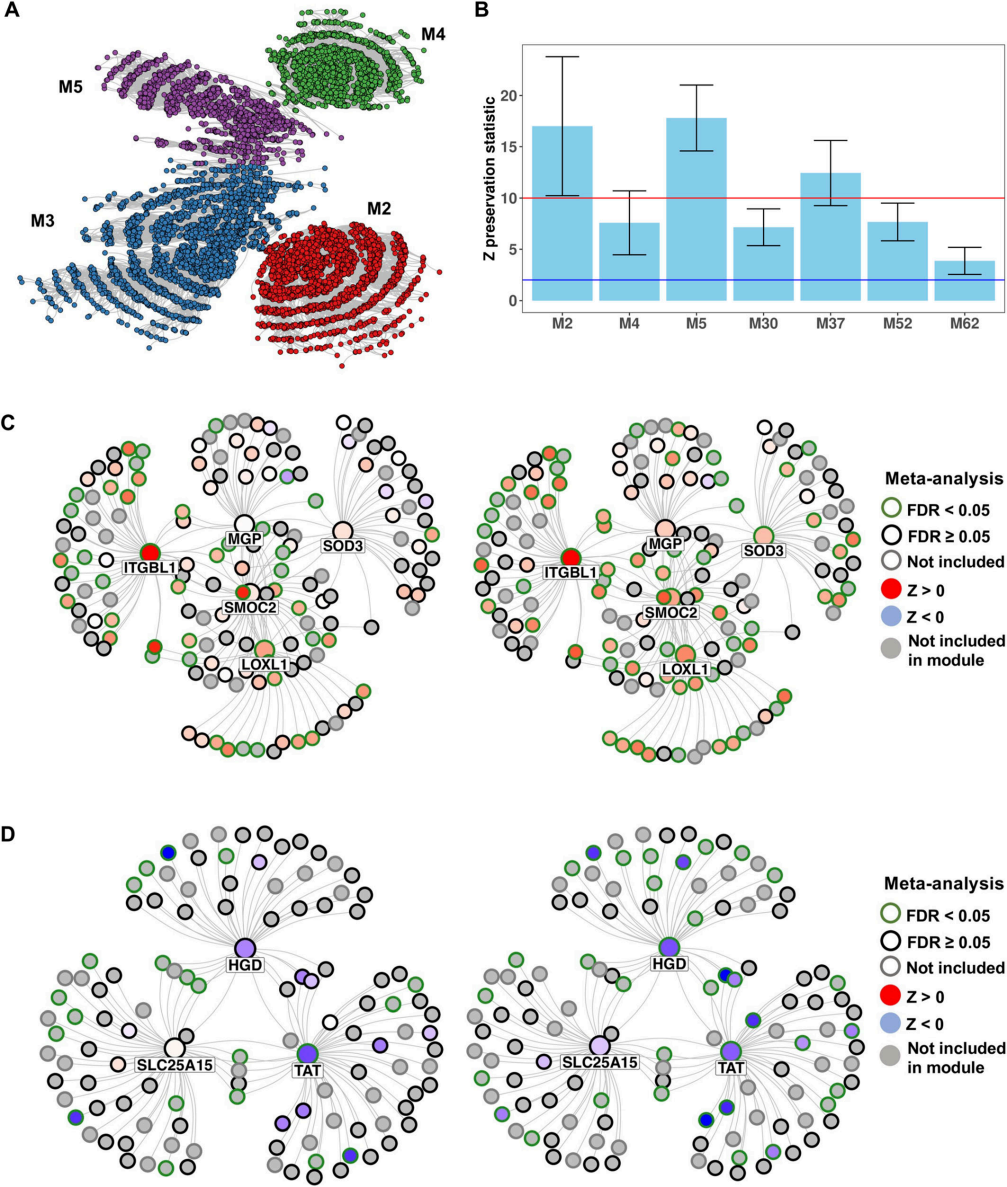
Coexpression, module preservation, and key driver analyses. **(A)** Comprehensive coexpression network color-coded by top-level modules M2, M3, M4, and M5. **(B)** Average preservation statistics across six validation datasets for the seven functionally relevant modules associated with MASH and MASLD. The blue and red lines indicate the preservation statistics cutoffs for moderate (2 ≤ Z < 10) and strong (Z ≥ 10) module preservation, respectively. **(C)** Bayesian causal subnetwork showcasing the top five key drivers along with their neighbors in module M5: MASH versus CTL and MASH versus MASLD. **(D)** Bayesian causal subnetwork showcasing the significant key drivers along with their neighbors in module M52: MASH versus CTL and MASH versus MASLD.

We first analyzed PRJNA512027 as a discovery dataset and included 8,262 variable genes, which led to the detection of 103 significantly coexpressed modules (*P* < 0.01 and comprising 50 or more genes). The entire coexpression network with the four top-level modules (M2, M3, M4, and M5) highlighted is depicted in Fig 5A. We extracted the eigengenes and conducted a differential expression analysis comparing MASLD versus CTL, MASH versus CTL, and MASH versus MASLD. In the comparison of MASH versus CTL, we identified 49 significant differentially expressed modules, and in MASH versus MASLD, we found 69 significant differentially expressed modules (Table S15). A total of 49 modules were differentially expressed in both MASH versus CTL and MASH versus MASLD comparisons. However, no differentially expressed modules were detected in the MASLD versus CTL comparison.

Table S15 Differentially expressed coexpression modules in MASH versus CTL and MASH versus MASLD.

M2 and M5 were among the top-level modules with the highest significance (down-regulated and up-regulated, respectively, in MASH versus CTL). In addition, M2 was significantly down-regulated in MASH versus MASLD, whereas M4 and M5 were significantly up-regulated in MASH versus MASLD. In the analysis of modules other than the top-level ones (i.e., M2-M5), we detected M52, M53, M37, and M62 in MASH versus CTL, and M52, M53, M37, M62, and M30 in MASH versus MASLD. The GO analysis of the significantly expressed top modules revealed significant GO enrichment in 55 modules, accounting for a total of 3,859 GO functional classes (Table S16). Among the top-level modules, M2 was enriched for ribosomal, oxidative phosphorylation, and ATP metabolic processes (hub gene: *HPN*); M4 for ion and gate channel activity (hub gene: *KRT77*) and M5 for extracellular matrix (hub gene: *SDC4*).

Table S16 GO analysis of the differentially expressed modules between MASH versus CTL and MASH versus MASLD.

### All functionally relevant modules were preserved across datasets

**Figure S9.**
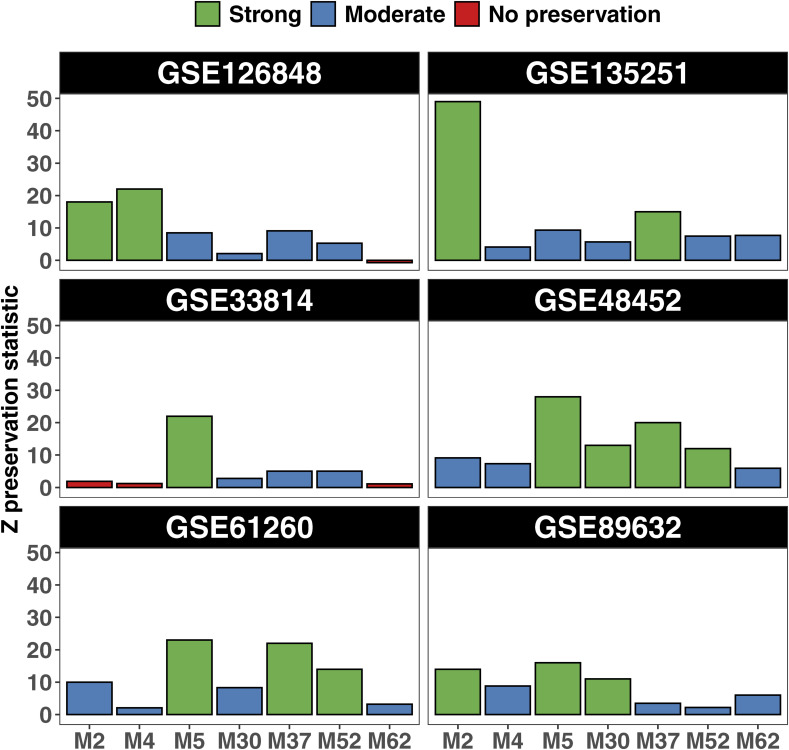
Module preservation statistics of the seven functionally relevant modules across the validation datasets.

We assessed the preservation of the seven functionally relevant modules M2, M4, M5, M30, M37, M52, and M62 across datasets GSE48452, GSE61260, GSE126848, GSE89632, GSE135251, and GSE33814. After averaging the preservation statistics across these datasets, we observed that all modules exhibited moderate to strong preservation in all datasets, with standard errors also falling in this range (Fig 5B). Specifically, modules M2, M5, and M37 showed strong average preservation, whereas all the other modules exhibited moderate preservation. Modules M2 and M4 showed no preservation in GSE33814, whereas module M62 showed no preservation in datasets GSE33814 and GSE126848 (Fig S9).

**Figure S10.**
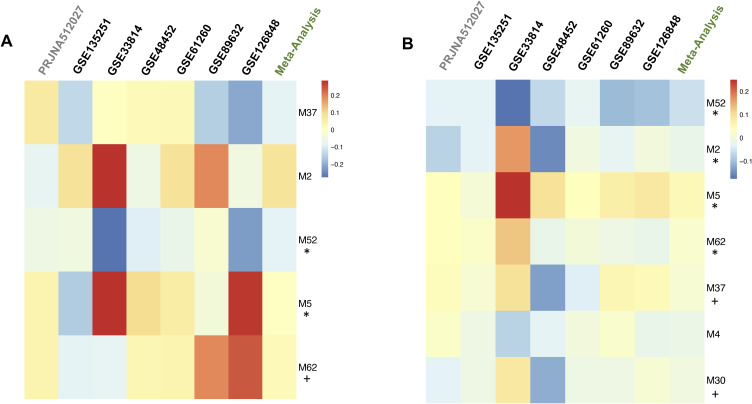
Validation of module differential expression. **(A, B)** The colors in the heatmap represent the log_2_ FC between MASH versus CTL (A) and MASH versus MASLD (B). The modules are ranked by meta-analysis adjusted p. * Module fully validated (same direction and statistically significant) + module partially validated (same direction but not statistically significant).

For validating module differential expression, we conducted a meta-analysis across the replication datasets. We designated modules as “fully validated” if they were statistically significant and exhibited concordant log_2_FC direction, and as “partially validated” if they were not statistically significant but showed concordant log_2_FC direction. We fully validated the differential expression of M52 and M5 in MASH versus CTL and partially validated module M62 (Table S17 (A) and Fig S10A). In addition, we fully validated the differential expression of modules M52, M5, M62, and M2 in MASH versus MASLD and partially validated module M30 in MASH versus MASLD (Table S17 (B) and Fig S10B). These results indicate that modules M2, M5, M52, and M62 are coexpression networks that are consistently and significantly associated with MASH and MASLD progression.

Table S17 Validation of coexpression modules in MASH versus CTL and MASH versus MASLD.

### Identification of key drivers in the coexpression modules

**Figure S11.**
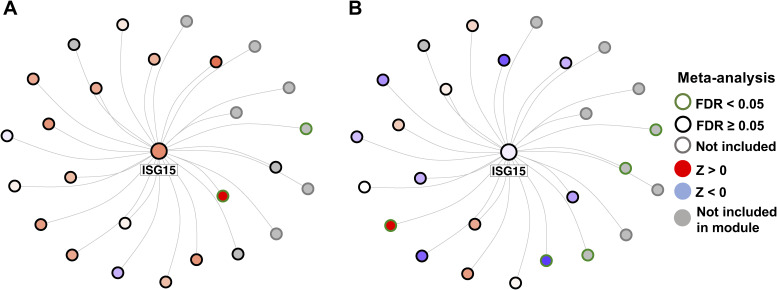
Bayesian causal subnetwork of M2 including the top key driver ISG15. **(A)** Comparison between MASH and CTL. **(B)** Comparison between MASH and MASLD.

**Figure S12.**
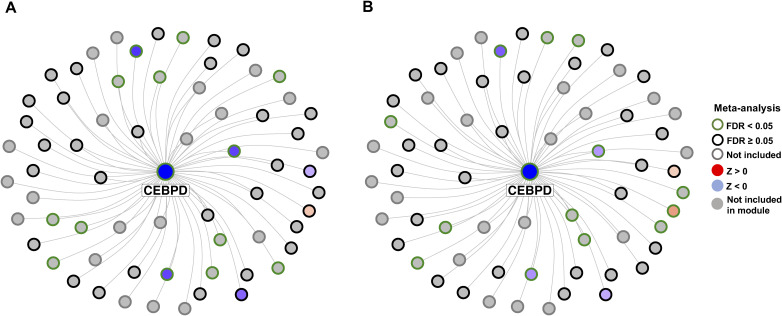
Bayesian causal subnetwork of M30 including the top key driver ISG15. **(A)** Comparison between MASH and CTL. (B) Comparison between MASH and MASLD.

We conducted a key driver analysis on the five fully and partially validated modules (M52, M5, M62, M2, and M30), identifying a total of 181 significant key drivers located within the coexpression modules (false discovery rate [FDR] < 0.05) (Table S18). The most significant key drivers (FDR < 2.2 × 10^−26^) were all located in module M5 and included SPARC-related modular calcium binding 2 (*SMOC2*), *ITGBL1*, lysyl oxidase like 1 (*LOXL1*), matrix Gla protein (*MGP*), and superoxide dismutase 3 (*SOD3*) (Fig 5C). Notably, *ITGBL1* and *LOXL1* were significantly overexpressed in the MASH versus CTL meta-analysis, whereas *SMOC2*, *ITGBL1*, *LOXL1*, and SOD3 were significantly overexpressed in the MASH versus MASLD meta-analysis (Fig 5C). Module M52, which was fully validated in both comparisons, yielded three significant key drivers: tyrosine aminotransferase (*TAT*), homogentisate 1,2-dioxygenase (*HGD*), and solute carrier family 25 member 15 (*SLC25A15*) (Fig 5D). *TAT* was significantly down-regulated in MASH versus CTL and significantly down-regulated in MASH versus MASLD, whereas *HGD* was significantly down-regulated in MASH versus MASLD. In contrast, the other fully validated module, M62, did not exhibit any significant key drivers. The causal networks for the partially validated modules M2 (top key driver: ISG15 ubiquitin like modifier [*ISG15*]) and M30 (top key driver: CCAAT enhancer binding protein delta [*CEBPD*]) are depicted in Figs S11A and B and S12A and B, respectively. *ISG15* was overexpressed in MASH versus CTL and in MASH versus MASLD, albeit not significantly (Fig S11A and B). Lastly, *CEBPD* was significantly down-regulated in MASH versus CTL and MASH versus MASLD (Fig S12A and B).

Table S18 Significant key drivers ranked by false discovery rate.

## Discussion

In this study, we present the outcomes of an extensive meta-analysis of hepatic gene expression data for MASLD and MASH, representing the most comprehensive analysis conducted to date. Our key discoveries include the identification of distinct gene expression patterns that distinguish MASLD, MASH, and healthy liver tissue. In addition, we validated two differentially expressed coexpression modules and identified novel key drivers with significant regulatory potential within these coexpression networks. Collectively, these findings provide new insight into potential liver-specific molecular mechanisms underlying the development and progression of MASLD.

To the best of our knowledge, there have only been two previously published meta-analyses investigating hepatic gene expression in MASLD. The first study used microarray-based data from seven GEO datasets, encompassing 137 MASLD samples, and identified a 218-gene signature associated with MASLD (31). Unlike the current work, this analysis correlated gene expression with clinical variables rather than disease status, analyzed data individually without merging for statistical analysis, and did not investigate hub genes or key drivers within coexpression networks. Consequently, there was minimal methodological overlap between the two investigations. The results from the current work thus build upon these findings by incorporating a significantly larger sample size—nearly 10 times greater—merging all datasets for statistical analysis and conducting association analyses for both MASLD and MASH diagnosis.

In the second study (32), a comprehensive analysis of 12 datasets comprising a total of 812 samples was performed to identify a gene expression signature specific to MASH. Although there were seven datasets that overlapped with our study, several notable differences emerged between the two analyses. First, the primary objective of the published analysis was to identify biomarkers associated with MASH, whereas our focus was on unraveling gene expression networks that could provide insights into key pathological mechanisms. Second, the published meta-analysis included datasets from pediatric MASLD studies, which may introduce potential confounding factors when combined with adult samples. Third, the previous analytical design involved segregating the datasets into Discovery (N = 309) and Validation (N = 503) groups, whereas we opted to integrate all datasets for our analysis. It is worth mentioning that most datasets within the Discovery group comprised fewer than 20 MASLD or MASH samples, which could impact the statistical power of the analysis.

Interestingly, some notable distinctions emerged in our results when comparing the DEGs identified in the MASLD and MASH meta-analyses. First, there was a substantial contrast in the number of DEGs between the MASH and CTL meta-analysis (N = 1,870) relative to the MASLD versus CTL analyses (N = 685). Moreover, the MASH versus MASLD meta-analysis identified 3,284 DEGs. The difference in the number of DEGs may reflect underlying biological variability between MASLD and MASH, suggesting that these two conditions, while related, have distinct transcriptomic signatures and potentially different underlying mechanisms. Alternatively, the greater number of DEGs in the MASH analysis might indicate that MASH is a more complex and heterogenous condition with a wider range of gene expression changes.

Second, the affected pathways differed depending on the analysis. In the MASLD analysis, the most significantly altered pathways included activin binding and receptor activity, glycogen biosynthesis, exocytosis, and ion transmembrane transporter activity. These results corroborate previous studies. For instance, dysregulated glycogen metabolism is recognized as a potential risk factor for MASLD (33), as are abnormal serum levels of activin (34). Exocytosis processes may potentially be linked to extracellular vesicles, which have been associated with the development of both whole-body and hepatic insulin resistance, as well as steatosis in the context of MASLD (35). In contrast, the MASH analysis detected functional classes associated with sulfur and alpha-amino metabolic processes and extracellular matrix organization. The DEGs from the MASH versus MASLD comparison were associated with the extracellular matrix, chemotaxis, catabolic processes of organic compounds, carboxylic acids, and alpha-amino acids. Interestingly, the same MASH-associated pathways were also identified in the coexpression analysis. The dysregulation in affected pathways again suggests that MASH and MASLD have different underlying disease mechanisms. For example, the involvement of pathways related to sulfur and alpha-amino metabolic processes in MASH might indicate a role for oxidative stress and amino acid metabolism in the pathogenesis of MASH, which is typically characterized by inflammation and fibrosis. In contrast, the pathways related to activin binding and receptor activity in MASLD may highlight the importance of certain signaling pathways in the earlier stages of liver fat accumulation.

We conducted a key driver analysis on the functionally relevant coexpression modules to identify genes with a disproportionately significant impact on the regulation of other genes within the fully validated M5, M52, and M62 modules. Whereas M62 exhibited no significant key drivers, both the M5 and M52 modules yielded noteworthy results. The M5 module was found to be significantly up-regulated in comparisons of MASH versus CTL and MASH versus MASLD and was enriched for “extracellular matrix” GO functional classes, consistent with the main gene meta-analysis. This finding is consistent with the development of hepatic fibrosis, primarily as a result of abnormal expression and accumulation of extracellular matrix proteins in the liver (36). In addition, we identified five significant key drivers with potentially critical regulatory roles. One of these genes, *SMOC2*, a member of the SPARC family of matricellular proteins, displayed increased hepatic expression in individuals with MASLD and mice fed a high-fat diet (HFD) (37). Notably, *SMOC2*-knockout mice exhibited protection against liver fibrosis and reduced hepatic inflammation induced by HFD (37). Larsen et al (38) recently observed elevated hepatic and plasma levels of *SMOC2* in individuals with MASH compared to those without MASLD and showed that *SMOC2* is primarily expressed by hepatic stellate cells, which play a pivotal role in fibrogenesis. *SMOC2* levels were also elevated in hepatocellular carcinoma tissue relative to normal tissue, and *SMOC2* overexpression promoted hepatocellular carcinoma cell proliferation (39).

In addition to *SMOC2*, the remaining key drivers identified in the M5 module have previously been linked with MASLD. In individuals with chronic hepatitis B, *ITGBL1*, which promotes cell migration (40), has been shown to regulate fibrogenesis (25) and is part of a six-gene signature predictive of cirrhosis (41). In a weighted gene coexpression network analysis (WGCNA) focused on susceptibility gene modules related to immune cells in MASLD, *ITGBL1* was among the hub genes linked to immune infiltration, fibrosis progression, and activity score (42). *LOXL1*, a member of the lysyl oxidase (LOX) family of enzymes involved in collagen and elastin crosslinking, has also been linked to liver cirrhosis (43, 44). Studies have demonstrated that down-regulation of *LOXL1* can slow disease progression (44), and HSC-specific *LOXL1* knockout in a mouse model of non-obese NASH attenuated liver steatosis, inflammation, and fibrosis, suggesting that LOXL1 may be involved in HSC activation and fibrogenesis (45). *SOD3*, which encodes an antioxidative enzyme with various functions (46), was found to promote HSC activation and fibrogenesis when deficient (47), whereas its overexpression in HFD-fed mice blocked the obesity, hepatic steatosis, and insulin resistance typically induced by this diet (48). MGP, an extracellular matrix protein that inhibits calcification (49), has only recently been implicated in MASLD. Research by Hui et al (50) showed that hepatic *Mgp* expression increased in tandem with MASH progression in a mouse model and was significantly correlated with fibrosis severity in humans with MASH (50). *Mgp* was highly expressed in HSC and identified as a key driver for liver fibrosis through network modeling, associated with the regulators of fibrosis, *Loxl1*, *Smoc2*, and *Itgbl1* (50).

The M52 module was significantly down-regulated in the MASH versus CTL and MASH versus MASLD comparisons and was enriched for alpha-amino acid metabolic process, consistent with the main gene meta-analysis. Altered levels of circulating amino acid levels have been observed in MASLD and MASH patients, along with differences in fatty acids and vitamins (51). *TAT*, *HGD*, and *SLC25A15* were identified as significant key drivers for this module. *TAT*, significantly down-regulated in both MASH meta-analyses, encodes a hepatic enzyme that catalyzes the conversion of tyrosine to 4-hydroxyphenylpyruvate. Mutations in *HGD* and *SLCA25A15* lead to the development of alkaptonuria (52) and hyperornithinaemia-hyperammonaemia-homocitrullinuria syndrome (53), respectively. Unlike the key drivers identified for the M5 module, there is currently no evidence connecting these genes to the development or progression of MASLD. These genes might therefore represent novel candidates for functional studies investigating the molecular features of MASH and MASLD associated with amino acid-related metabolic dysfunction. The relevance of these results is strengthened by the consistent patterns found across several datasets.

Our comprehensive study design and bioinformatics workflow enabled us to identify robust and consistent signals at the network level across different datasets, pinpointing key genes that might provide insights into new therapeutic targets and further define the molecular landscape of MASLD and MASH. Despite the strengths of our rigorous approach, we acknowledge certain limitations. First, while RNA-sequencing provides a comprehensive assessment of a tissue’s transcriptome, integrating microarray studies into the meta-analysis, with their more limited genome coverage, presents a risk of missing significant genes. This occurs because the analysis depends on genes that are common to all datasets. Nevertheless, we adopted this approach to include a wider range of datasets, thereby enhancing the robustness of our findings. Second, it’s important to note that we did not conduct any experimental validation of the detected key drivers and their perturbed networks. Although consistent signals were observed, further investigation is necessary to confirm the regulatory role of these key drivers in both in vitro and in vivo models, as well as determine their potential effect on MASLD and MASH pathology.

Our study provides a comprehensive and robust view of hepatic gene expression changes associated with MASLD and MASH. The variations in the number of DEGs detected in the individual MASH and MASLD analyses suggest that these two conditions are not only phenotypically different, but also exhibit distinct molecular profiles. Further research into these differences can potentially lead to a deeper understanding of the conditions and improved clinical management strategies. Our findings underscore the importance of meta-analysis in elucidating complex disease processes and highlight the need for additional investigations to validate and expand upon these results.

## Materials and Methods

### Data preprocessing

The data selection workflow is illustrated in Fig S1A. Criteria and results of the search are provided in the results section. We executed the preprocessing of raw data using separate pipelines for RNA-seq and microarray data. The details of the workflow are illustrated in Fig S2B. For RNA-seq data (GSE135251, GSE126848, GSE130970), we obtained raw data files from sequencing read archives and performed alignment with the GRCh38 human genome reference using *kallisto 0.46.1* (54). We imported transcript-level abundance using Entrez Gene annotations (55) and estimated counts and transcript lengths with the *txtimport* package (56) to obtain a matrix of average transcript length, weighted by sample-specific transcript abundance estimates, to counterbalance different expressions of gene-level counts. We then excluded genes with fewer than five total counts across all samples and applied a variance-stabilizing transformation via the *vsd* function from DESeq2 (57), which transforms the count data and provides an approximately homoscedastic matrix of values. Principal Component Analysis was used to identify and remove outlier samples, defined as those exceeding the cutoff of ±4 standard deviations from the mean on at least one of the top two principal components. Raw counts were normalized with the *voom* method (58), and surrogate variable analysis was conducted via the *sva* function with the *leek* method from the *sva* R package (59). The derived surrogate variables were used to adjust the *voom*-normalized expression values using the *removeBatchEffect* function from *limma* (60). Gene annotations were standardized across datasets to HGNC (HUGO Gene Nomenclature Committee) symbols using the *R-BiomaRt* package (61).

For the preprocessing of Affymetrix microarray data (GSE167523, GSE83452, GSE61260, GSE48452), we obtained raw data (CEL files) from GEO and normalized the data using the Robust Multi-Array Average algorithm (62), as implemented in the *oligo* R package (63). We used the *arrayQualityMetrics* R package (64) to evaluate data quality with respect to reproducibility, outliers, and signal-to-noise ratio. We generated and analyzed several visualizations, including heatmaps depicting inter-array expression distances, Principal Component Analysis plots, and MA-plots (using log-intensity ratios and log-intensity averages), which enabled us to identify patterns, trends, and potential outliers within the data. Samples were classified as outliers in two of the three metrics in the initial round of QC runs from the dataset and were subsequently removed from further analysis. After outlier removal, data were once again normalized starting from the raw data, excluding genes in the lower 25th percentile of average expression and adjusting for surrogate variables, thus mirroring the workflow applied to the RNA-seq data. Gene annotations were standardized as described above. For Illumina microarray datasets (GSE33814, GSE89632), we downloaded the matrix of normalized expression values from GEO and applied the same workflow as described for Affymetrix data, excluding the normalization step (Fig S1B).

### Gene expression meta-analysis

We used the methodology of Choi et al (23) to generate a comprehensive ranked gene list based on the FDR associated with each gene. The input consisted of the *voom*-normalized (for RNA-seq) and RMA-normalized matrices (for microarrays), adjusted for surrogate variables. We executed the analysis using the R package *GeneMeta* setting a random-effects meta-analysis model to account for heterogeneity across studies and standardizing data using Z-score transformation (function: “*Zscore*”). The FDR for each gene was generated using the “*ZscoreFDR*” function, with 50,000 permutations factored into the calculations. We considered an FDR < 0.05 to be statistically significant evidence for differential gene expression. Subsequently, we performed pathway analysis with the DEGs, with reference to the Gene Ontology (GO) database using the *enrichGO* function, as implemented in the *clusterProfiler* package. *P*-values were adjusted using the FDR method, and GO processes with an FDR < 0.05 were considered statistically significant.

### Enrichment with GWAS and TWAS results

We used four large GWAS to investigate the enrichment of MASLD-associated genes across our meta-analysis results. In addition, using the same GWAS, we investigated the relationship between genetically regulated gene expression and MASLD, conducting a TWAS imputing expression values from the *GTEx v8* liver expression data. The first GWAS involved the UK Biobank (UKB) cohort, which comprised 28,396 individuals with MASLD and 108,652 healthy individuals (29). MASLD status was determined using ICD codes, whereas a liver fat percent <5% by abdominal MRI defined controls. The second GWAS (28), also using the UKB cohort, included 4,761 MASLD cases and 373,227 unaffected controls. In this study, the authors assigned MASLD status using the diagnostic codes recommended by recent consensus guidelines. The third GWAS (30) included 1,106 MASLD cases and 8,571 controls and histological data from liver tissue in 235 available participants from the electronic medical records and genomics network. The samples were selected using a natural language processing algorithm billing codes, text queries, laboratory values, and medication records. Finally, the fourth GWAS (27) included 1,483 MASLD cases, and 17,781 controls histologically characterized. We obtained GWAS summary statistics for all the studies from the GWAS catalogue (Accession numbers: GCST90094908, GCST90054782, GCST008471, and GCST90011885, respectively). To ensure data quality, we performed data cleaning using the “*munge_sumstat*” function from *Linkage Disequilibrium Score Regression* (*ldsc*) software (65). During the data cleaning process, we conducted quality control checks and applied filters to include only relevant SNPs. Specifically, we retained SNPs with an imputation quality (INFO) greater than 0.9, a minimum allele frequency higher than 1%, and P-association values between 0 and 1. We also removed insertion/deletion variants (INDELs) and SNPs with duplicated “rs” numbers.

Subsequently, GWAS summary statistics were analyzed using the Multi-marker Analysis of GenoMic Annotation (MAGMA) method, which provides gene-level statistics using a multiple regression approach to incorporate linkage disequilibrium (LD) information between markers and to detect multi-marker effects (66). *P*-values were adjusted for multiple testing using the Bonferroni method, accounting for the number of genes tested. We then used the *FUSION* software to conduct the TWAS (67). We imputed expression values using liver data models from GTEx v8 (European reference data), which provide a relationship measure between an individual’s genotype and gene expression levels, thereby capturing the *cis*-acting genetic effects on gene expression. Using *FUSION*, we computed gene weights that estimate the effects of individual SNPs on gene expression. These gene expression models were then used to evaluate whether the predicted gene expression levels were associated with the phenotype. To account for multiple testing, we adjusted TWAS *P*-values using the FDR method.

We also performed an enrichment analysis to assess the functional relevance of the genes identified through MAGMA and TWAS (FDR < 0.05). In this analysis, we used the TWAS and MAGMA gene lists as the gene sets of interest and the DEGs from our meta-analysis as the candidate list. The reference set was the comprehensive list of genes included in each meta-analysis. To perform the enrichment analysis, we used the “enrichment” function from the bc3net R package. This function allows for the calculation of hypergeometric statistics, which assess the enrichment of the MAGMA and TWAS genes within the candidate list of DEGs.

### Coexpression analysis, key driver analysis, and module validation

Coexpression analysis was conducted using the Multiscale Embedded Gene Expression Network Analysis (MEGENA) algorithm, which offers a robust alternative to existing coexpression network clustering methods, including WGCNA (68). We selected the dataset PRJNA512027 as a discovery cohort as it encompasses MASH (n = 104), MASLD (n = 49), and CTL (n = 36) samples, and includes the largest sample sizes among the datasets available. We used other datasets that also included MASH, MASLD, and CTL for module preservation analysis. The matrix of SV-adjusted expression values was filtered to include only the top 50% of genes with the highest median absolute deviation. Network generation was executed using the MEGENA R package (68) based on the following workflow. Initially, we calculated signed pairwise gene correlations using Pearson’s method with 1,000 permutations, retaining correlations that were significant at the 5% FDR level (function: *calculate. correlation*). Significantly correlated gene pairs (FDR < 0.05) were ranked and iteratively tested for planarity, leading to the development of a planar filtered network using the planar maximally filtered graph technique (function: *calculate.PFN*). Subsequently, we conducted a multiscale clustering analysis to identify coexpression modules at varying network scale topologies and their respective hub genes (function: *do.MEGENA*). Coexpression modules deemed significant (with a permuted *P* < 0.01 and module of 50 genes or more) were carried forward for further analysis. Next, we extracted module eigengenes (the first principal component of the gene module) using the function *moduleEigengenes* from the *WGCNA* R package (69). Pairwise differential expression between diagnostic groups was computed using the *limma* R package (60). Modules with significant associations were annotated for GO functional classes, following the same workflow we adopted for DEGs.

To validate our findings, we conducted a module preservation analysis on the functionally relevant modules. We used the “*modulePreservation*” function from the WGCNA R package to derive the Z-score preservation statistics. Modules with a Z-score greater than 10 were deemed to have strong preservation, those with a Z-score >2 and <10 indicated moderate preservation, and those with a Z-score <2 showed no preservation. This preservation analysis was applied across datasets that included MASLD, MASH, and CTL samples (GSE48452, GSE61260, GSE126848, GSE89632, GSE135251, and GSE33814). Subsequently, we extracted the eigenvalues of the functionally relevant modules from the six validation datasets and then evaluated their differential expression, focusing on the comparisons: MASH versus CTL and MASH versus MASLD. The *P*-values and the log_2_ FC obtained from these validation datasets were combined through a meta-analytical approach based on the Fisher’s weighted test (70). The input was the unadjusted *P*-values derived from the differential module expression analysis. Given that the Fisher Z weighted test requires one-tailed *P*-values, we converted the two-tailed nominal *P*-values to one-tailed *P*-value. If the Log_2_FC was greater than zero, the formula used was: p1Tailed = p2Tailed/2. Otherwise, the formula was: p1Tailed = 1 − (p2Tailed/2). The uncorrected *P*-values were then weighted by the sample sizes of the datasets and combined with the *combine.test* function with the “*z.transform*” option, as part of the *survcomp* R package (71). We calculated the average log_2_ FC by weighting for sample size for each study using the “weighted.mean” R functions.

We applied weighted Key Driver Analysis to identify central hub genes within functionally relevant modules using the mergeomics R package. Hub genes are characterized by a high number of strong correlations with other genes in the network; however, this does not necessarily indicate a causal relationship. For this analysis, we used a liver-specific Bayesian regulatory network as a reference (72). The parameters set for the analysis were as follows: “search depth” was set to 1, “edge type” was specified as undirected, “minhuboverlap” was set at 0.33″ and “edge.factor” was 0. This algorithm prioritizes module genes based on their ability to regulate other genes in the module. It uses a pre-built causal regulatory network, identifying genes whose neighbors predominantly belong to the same coexpression module. These “key driver” genes, predicted as top regulators within a coexpression module, are targets for potential novel treatment designed to prevent the progression.

## Supplementary Material

Reviewer comments
